# Boronic acid based dynamic click chemistry: recent advances and emergent applications

**DOI:** 10.1039/d0sc05009a

**Published:** 2020-12-17

**Authors:** Saurav Chatterjee, Eric V. Anslyn, Anupam Bandyopadhyay

**Affiliations:** Biomimetic Peptide Engineering Laboratory, Department of Chemistry, Indian Institute of Technology Ropar Punjab-781039 India anupamba@iitrpr.ac.in; Department of Chemistry, University of Texas 1 University Station A1590 Austin Texas 78712 USA anslyn@austin.utexas.edu

## Abstract

Recently, reversible click reactions have found numerous applications in chemical biology, supramolecular chemistry, and biomedical applications. Boronic acid (BA)-mediated *cis*-diol conjugation is one of the best-studied reactions among them. An excellent understanding of the chemical properties and biocompatibility of BA-based compounds has inspired the exploration of novel chemistries using boron to fuel emergent sciences. This topical review focuses on the recent progress of iminoboronate and salicylhydroxamic–boronate constituted reversible click chemistries in the past decade. We highlight the mechanism of reversible kinetics and its applications in chemical biology, medicinal chemistry, biomedical devices, and material chemistry. This article also emphasizes the fundamental reactivity of these two conjugate chemistries with assorted nucleophiles at variable pHs, which is of utmost importance to any stimuli-responsive biological and material chemistry explorations.

## Abstract 2

Fundamental progress, current developments, and rapidly growing applications of iminoboronate and salicylhydroxamic–boronate conjugate esters are deliberated.

## Graphical abstract

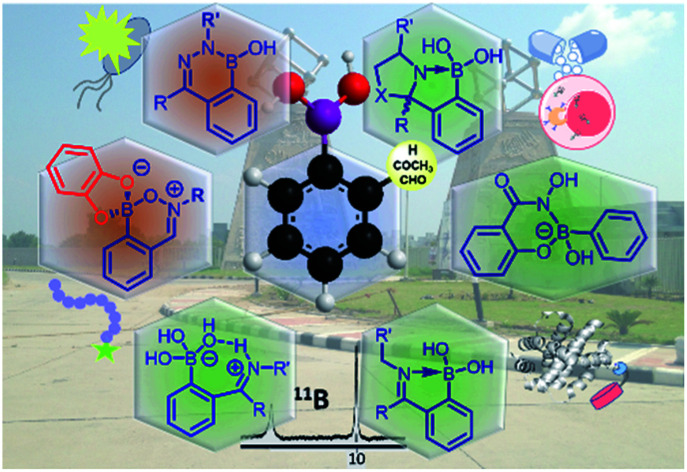

## 1. Introduction

**Fig. 1:**
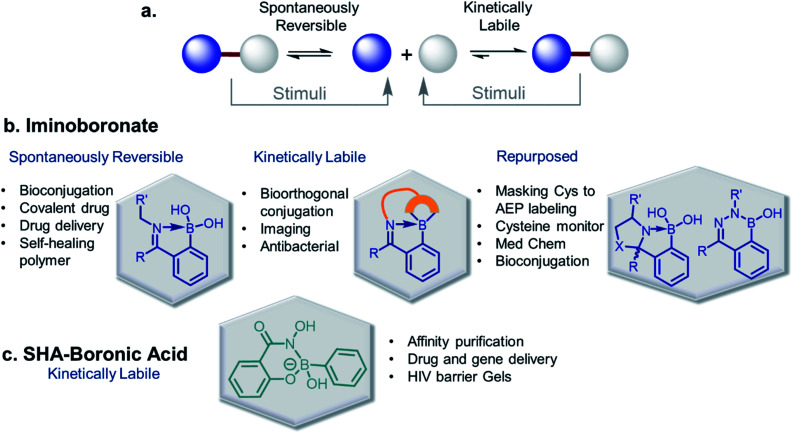
(a) A cartoon representation of dynamic covalent chemistry; application revealed with (b) iminoboronate and (c) SHAB conjugates.

The discovery of dynamic covalent chemistry (DCvC) has led to numerous applications in multidisciplinary sciences. Of these, spontaneously reversible or quickly exchangeable reactions have been applied to the creation of structural and functional components through constitutional dynamic chemistry^1^ and dynamic combinatorial chemistry.^2^ This methodology has been primarily applied to generating receptor- or “substrate driven” molecular recognition, finding bioactive substances, and to the development of dynamic materials.^2^ Capturing endogenous nucleophiles *via* spontaneous covalent conjugation has also been shown to be privileged in high-affinity drug discovery.^3^ In contrast, products that enjoy high thermodynamic stability, and yet are kinetically labile, are superior in the preparation of many bioconjugate applications. Despite their potential uses, reversible click chemistries are relatively overlooked in bioconjugation purposes. However, stimuli-responsive DCvC has found use in drug delivery and smart material innovation. An overview of spontaneously reversible and kinetically stable products is shown in Fig. 1a.

There are several classes of DCvC. Among them, α-nucleophile conjugates with carbonyl compounds, as well as reversible disulfide formation are noteworthy. They have been frequently used to generate dynamic combinatorial libraries and stimuli-responsive biomaterials, especially for the controlled drug release from antibody–drug conjugates.^7^ Numerous applications can be found in the previously documented reviews.^5–9^ However, these two classes of DCvC chemistries suffer from slow reaction kinetics under physiological conditions. Thus, several advancements *via* new conjugate partners (non-boronic acid) and external catalysts have been implemented to enhance the rate of α-nucleophile conjugations. These advances, however, are still not entirely suitable for *in vivo* applications.^8,10^ Yet, as discussed herein, an *ortho*-boronic acid to benzaldehyde or acetophenone can self-catalyze the reaction of α-nucleophile conjugations, increasing the rates a few orders of magnitude higher than their non-boronic acid counterparts.

In the toolbox of existing dynamic reactions,^2,7^ boron-employed DCvC has emerged as one of the most prevalent, ranging from catalysts^11^ to designer drugs, as well as probes in chemical biology and optoelectronics in material science.^12,13^ Importantly, boron chemistry allows conjugations that are dynamic at physiological pH. An aryl or alkyl BA features a planar trigonal structure attached to a boron atom possessing a vacant p-orbital. Further, with specific designs, they can form a dative bond with Lewis bases and reversible covalent bonds with oxygen and nitrogen containing nucleophiles at physiological pH.^13^ These properties can manifest themselves as catalytic properties of the BA, making BAs a unique moiety in multipurpose, adaptable applications. On this subject, the applications of BA-mediated *cis*-diol conjugation have been archived previously in numerous reviews,^11,13^ and therefore are not considered in this review.

Over the past ten years, the use of rapid imine formation and stabilization assisted by BAs *via* the creation of iminoboronates (Fig. 1b) has generated several applications. In addition, the BA-mediated salicylhydroxamic–boronate (SHAB) conjugate has numerous promising applications. Here, we discuss the considerable progress and resulting applications (Fig. 1b), primarily over the last five years of these two BA employed reversible covalent click chemistries. Further, the underlying mechanism of reversibility, kinetics, conjugation partners, structural activity, and scopes of both conjugates (Fig. 1) are critically discussed.

## 2. Iminoboronate chemistry

**Fig. 2:**
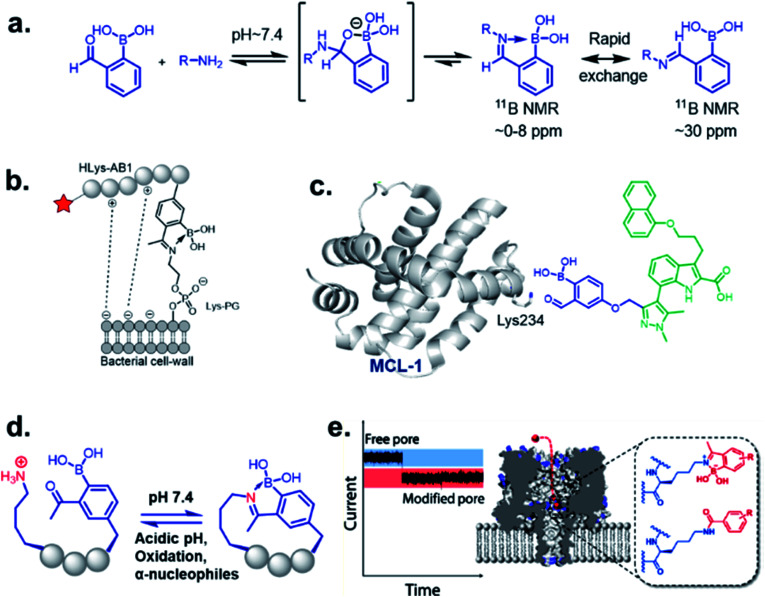
(a) Mechanism of iminoboronate conjugation, and applications revealed with spontaneously reversible iminoboronate formation; (b) labelling of the bacterial membrane, (c) structure-based covalent drug design for targeting MCL1, (d) spontaneous peptide cyclization that responds to stimuli, (e) an alternative to genetic modification of membrane nanopore through ionic current measurement. Reproduced with permission from *ACS Nano*, 2018, **12**, 1, 786–794, Copyright (2018) American Chemical Society.

Stable imine or Schiff base formation in anhydrous organic solvents is well known. Generally, a classical Schiff base suffers from thermodynamic instability in an aqueous environment. However, the addition of a BA moiety at the *ortho* position of benzaldehyde (*i.e.* 2-formylphenyl boronic acid (2-FPBA)) or acetophenone (*i.e.* 2-acetylphenyl boronic acid (2-APBA)) markedly improves the thermodynamic stability over a pH range of 6–10 *via* iminoboronate formation. Mechanistically, the BA speeds up the rate-determining dehydration step, and in many cases is proposed to create a Lewis conjugate (N → B dative bond) that is thermodynamically stabilizing. However, the purported Lewis acid/base complex considerably polarizes the imine bond to facilitate quick product hydrolysis (Fig. 2a) or imine scrambling with another amine. Thus, the BA creates a kinetically more labile imine bond in a reversible process while also imparting improved thermodynamic stability (∼3–4 kcal mol^−1^ extra for N→B dative bond and overall product stability ∼ 10 kcal mol^−1^ (ref. 12)) in comparison to a classical Schiff base.

**Scheme 1:**
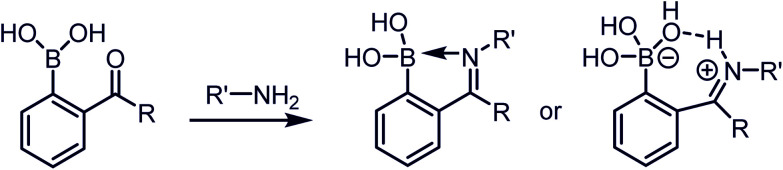
The proposed iminoboronate structure.

A dative N → B bond is depicted in the chemical structures throughout this review because this is the interaction drawn in the original literature by the primary authors of the studies. However, we note here that the Anslyn and James groups have performed structural analyses of iminoboronate complexes *via*^11^B-NMR spectroscopy. They concluded that the studies did not support dative interactions.^14^ Instead of a dative bond, the ^11^B-NMR chemical shifts were interpreted to support solvent insertion between the N and B atoms (Scheme 1). Similar to a dative interaction, solvent insertion would also create a kinetically labile imine bond because it is protonated at neutral pH, while also imparting considerable thermodynamic stability due to the intramolecular contact ion-pair. Further, some of the studies reviewed herein create aminoboronate complexes subsequent to iminoboronate formation. Aminoboronate complexes are well-accepted to be dominated by solvent-insertion in protic media.^15^ Importantly, each FPBA and APBA derived iminoboronate or aminoboronate systems can exist either with a N → B dative bond or with a solvent inserted, and structural analysis *via*^11^B-NMR spectroscopy and crystallography can be performed to resolve the issue when it is critical to a full understanding of the chemistry.^16^

An iminoboronate complex was first reported by Dunn *et al.* in 1968.^17^ Thereafter, the James and Bull groups have shown fruitful utilization of this chemistry through chiral amine resolution in NMR,^18^ and host–guest assembly in organic solvents. However, the Gois group was the first to show that this chemistry can be used for protein lysine side chain modification under physiological conditions, providing a clean and highly efficient ‘click reaction’.^4^ They also examined the reaction mechanism through density-functional theory (DFT), as well as product reversibility with the addition of small molecule competitors such as dopamine, glutathione, and fructose. This milestone paper has led to the expansion of this chemistry into many multidisciplinary science areas. For purposes of this review, we have categorized iminoboronate chemistry into three different types (Spontaneously reversible, kinetically labile, repurposed) and limited our examination to advancement in the last five years; discussing basic principles of the underlining chemistry and highlighting suitable applications.

### 2.1 Spontaneously reversible

It has been demonstrated by Bandyopadhyay *et al.* that the iminoboronate formation is reversible with a *K*_d_ ∼ 10 mM under physiological conditions, and the reversibility does not require small molecule competitors.^19^ Dilution of an iminoboronate complex between 2-APBA and lysine spontaneously dissociates into the reactants.^20^ An understanding of the molecular mechanism allow its implementation to realize the selective recognition of amine-containing biomolecules of interest in complex biological systems.

One of the most remarkable utilization of this chemistry in labelling of bacterial pathogens, was shown by Bandyopadhyay *et al.*,^19^ where a 2-APBA moiety attached to a cationic peptide selectively labelled *S. aureus* overexpressing Lys–PG on the outer leaflet of cell membranes. The probe evaded mammalian cells and other possible contenders for iminoboronate formation, such as serum proteins, while attaining selectivity from the electrostatic attraction between the cationic peptide and the anionic membrane (Fig. 2b). Using a similar strategy, they have recently designed peptide probes which bind to colistin-resistant bacteria primarily by targeting PE-modified lipids.^21^ The probes were reportedly acquired by screening a chemically modified phage library against colistin-resistant pathogens. The reversibility of the iminoboronate conjugate enables the final product to exchange with other amines, a desirable feature for biomolecular recognition, avoiding off-target modification. Irreversible covalent drug design sometimes suffers from off-target modification, a shortcoming that is resolved by the exchangeable feature of iminoboronates, leading to the suitably applied reversible covalent inhibition of a protein target, demonstrated by Akçay *et al.* They have shown that small molecule inhibitors of Mcl-1 (an oncogenic target) attached to 2-APBA or 2-FPBA probe exhibits 20–50 times better binding affinity (*K*_d_ = 3 nM) compared to control molecules.^22^ The probe was implemented to capture a non-catalytic lysine side residue near the small molecule binding pocket of Mcl-1 (Fig. 2c). An interesting application was further explored by Bandyopadhyay *et al.* through a spontaneous, stimuli-responsive peptide macrocyclization.^20^ A 2-APBA moiety can be used as an intramolecular conjugate partner of a lysine side chain in any particular peptide sequence. This concept was utilized to build unique macrocyclic and bicyclic peptides that respond to pH and oxidation (Fig. 2d). This type of peptide cyclization technology could be applied to stimuli-responsive biomaterial science or the preparation of cyclic peptide libraries. Another application was revealed through the alternative genetic modification approach of membrane nanopores, demonstrated by the Cockroft group (Fig. 2e). Using the reactivity kinetics of lysine side chains with 2-APBA in the nanopore system by ion-currents, a specific lysine side chain could be modified for further applications.^23^

**Fig. 3:**
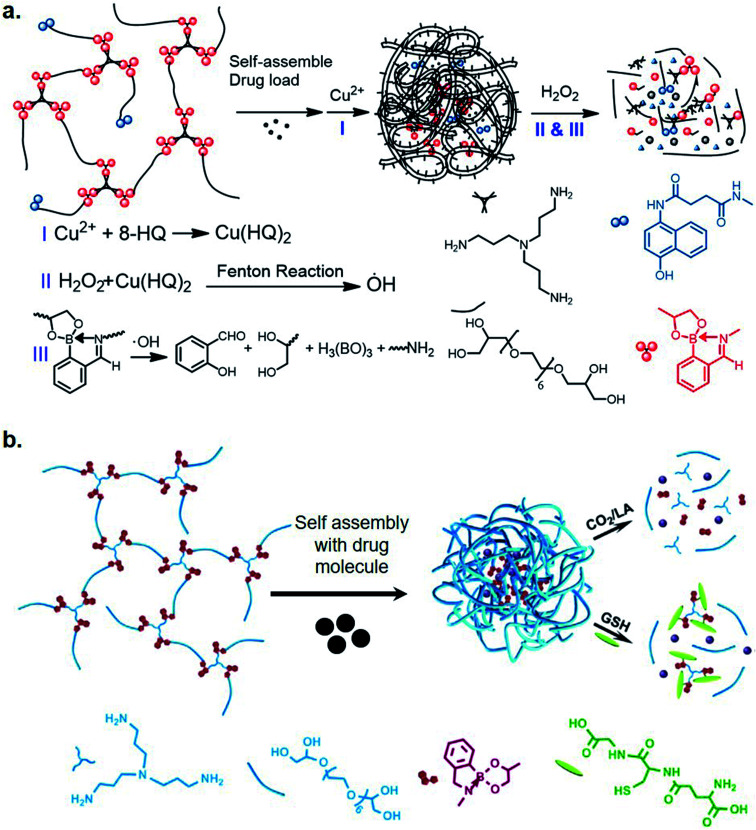
Iminoboronate-based stimuli-responsive biomaterials: (a) ROS responsive nanocarriers for cancer therapy, and (b) a triple-stimuli backbone-breakable polymer that responds exposure to CO_2_, lactic acid, and GSH. Reproduced with permission from *Chinese Chemical Letters*, 2020, **31**, 7, 1822–1826, Copyright (2020) with permission from Elsevier.

Recently, Zhang *et al.* utilized iminoboronate chemistry in designing ROS-responsive nanocarriers for cancer therapy.^24^ They designed polymeric nanocarriers of an iminoboronate backbone-based hyperbranched polymer with 8-hydroxyquinoline moieties (HBP(OEG-IB)-HQ) for binding metals. Complexation of Cu(ii) ions and the HQ moieties generated HQ–Cu catalytic sites where a Fenton-like reaction between H_2_O_2_ and HQ–Cu, generated OH radicals, which caused oxidative cleavage of iminoboronate moieties (Fig. 3a). This effectively disrupted the nanocarriers, leading to a rapid release of encapsulated drugs. They also designed a Cu(ii)-mediated Fenton reaction-enhanced ROS-response therapeutic polymersome, a metallisable triamine-centered iminoboronate functionalized amphiphilic starlike prodrug (N_3_-(OEG-IBCAPE)_4_) with four oxidation-cleavable iminoboronate moieties (IBCAPE). The accelerated oxidation of ROS moieties of iminoboronatres by Cu(ii) complexed vesicles, leads to an improved release of parent CAPE molecules. The release profile was found to spatially match the location of generated ROS in HepG2 cells.^25^ Prior to this study, Liu *et al.* had also developed triple-sensitive polymer nano-aggregates *via* iminoboronate formation using methoxypolyethylene glycol amine, 2-FPBA, and bis(2-nitrophenyl) ethanediol (a photo-cleavable nitrobenzyl alcohol derivate)^26^ in an aqueous medium, emphasizing applications in drug and gene delivery. Recently, the same group designed a triple-stimuli responsive backbone-breakable polymer (HBP(OEG-IB)) of oligo(ethylene glycol), tris(3-aminopropyl)amine and 2-FPBA.^27^ The amphiphilic nontoxic micelles formed by (HBP(OEG-IB)) demonstrated the release of camptothecin upon exposure to CO_2_, lactic acid, and GSH (Fig. 3b) in an *in vitro* study with HeLa cells. They proposed that the micelles enter the lysosomes and then permeate into the nucleus of the tumor cells as the mode of action.

**Fig. 4:**
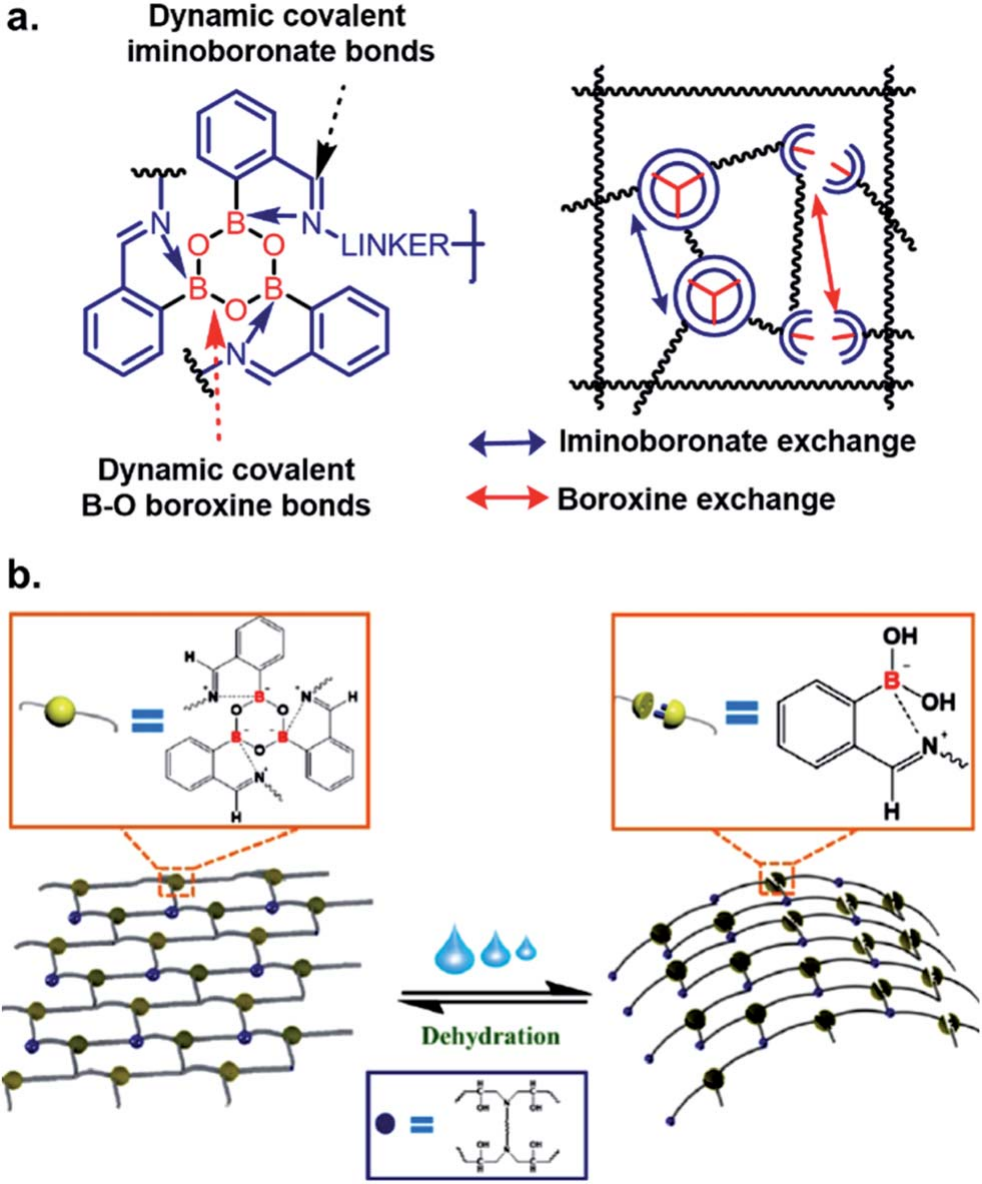
Iminoboronate based (a and b) self-healable polymer. Reproduced with permission from *ACS Appl. Mater. Interfaces* 2019, **11**, 19, 17853–17862, Copyright (2019) American Chemical Society.

In the field of material science, the applicability of iminoboronate chemistry has perhaps been best applied to self-healing polymers,^28,29^ where dynamic boroxine/BA equilibrium and iminoboronate chemistry was used to construct polymeric materials capable of self-healing without requiring any energy-demanding external activation (Fig. 4a). The self-healable and mechanically adaptive nature of sea cucumber inspired the Manas-Zloczower group^30^ to synthesize a water-responsive self-healing polymer by cross-linking poly(propylene glycol) with boroxine and epoxy (Fig. 4b). Dynamic boroxine formation with 2-FPBA provided the self-healing property whilst maintaining tensile modulus and stress on par with classic thermosets. Changes from a rigid thermoset to a soft rubber upon addition of water indicates possible applications to transforming printing for micro/nanofabrication. These versatile polymers also have possible applications in wearable electronics for human healthcare. A gas-controllable lower critical solution temperature (LCST)-type hyperbranched polymer with CO_2_-hydrolyzable iminoboronates was developed with potential applications in both biomedical and material chemistry.^31^

Stimuli-responsive hydrogels are suitable for various bioengineering purposes. They expand the choice of suitable materials, while limiting the complexity of the synthesis, and diminishing the undesired drug-release behavior of typical hydrogels. Recently, the Shi^32^ and Das^33^ groups have utilized iminoboronate chemistry to develop self-healable hydrogels with possible applications to wound healing and pH-responsive drug delivery.

**Fig. 5:**
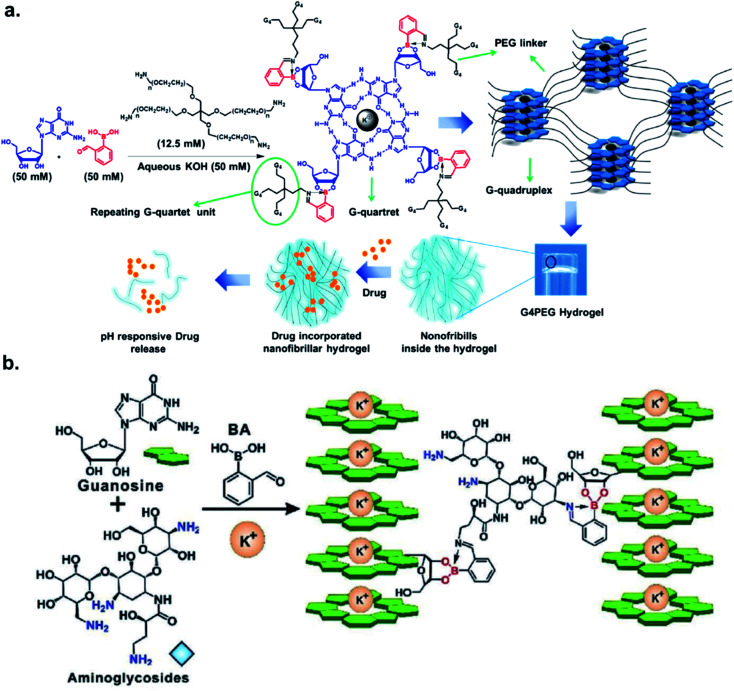
Iminoboronate based multistimuli responsive biopolymers for (a) doxorubicin delivery to cancer cells. Reproduced with permission from *ACS Appl. Bio Mater.*, 2020, **3**, 2, 1052–1060, Copyright (2020) American Chemical Society; and (b) killing bacteria. Reproduced with permission from *Advanced Healthcare Materials*, 2019, **9**, 2, 1901329, Copyright (2020) John Wiley and Sons.

The Das group engineered and characterized a dynamic G-quadruplex hydrogel using guanosine, 2-FPBA, and 4-Arm PEG-NH_2_ (Fig. 5a). Rheological experiments revealed a 90% retention of mechanical strength after four cycles. They also incorporated the anti-cancer drug doxorubicin in the hydrogel, and measured a drug release rate of 5.93 × 10^−5^ μmol s^−1^ at pH 4.8 *vs.* 2.27 × 10^−5^ μmol s^−1^ at pH 7.4 in the MCF-7 cell line.^33^ The authors demonstrated the faster loss of iminoboronate cross-linking in acidic pH with a nearly zero-order drug release profile. Zhang *et al.* reported the synthesis of a multistimuli-responsive hydrogel with potent antitumor activity.^34^ Brilliant utilization of 2-FPBA, K^+^, aminoglycoside, and guanosine enabled the formation of guanosine quadruplexes, which could be connected to aminoglycosides, a class of potent antibiotics, *via* 2-FPBA as the linker (Fig. 5b). As a result, an all-small-molecule supramolecular assembly could release the antibiotics upon exposure to heat, acids, oxidants, glucose, and crown ethers. The aforementioned reports highlight the popularity of iminoboronate chemistry in the application to biopolymer endeavours.

Another development in iminoboronate chemistry was an improvement in the thermodynamic stability of the iminoboronate product by introducing better nucleophiles. In this regard, 2-APBA demonstrates quick reversibility but better thermodynamic stability with hydrazides (*K*_d_ ∼ 0.6 mM), and oxyamines (14 μM)^35^ in comparison to amines. The conjugate of 2-FPBA-hydrazide and oxyamine also showed similar properties (Table 1). 2-FPBA-benzyloxyamine was found to be highly stable, lacking a N–B dative or a distant dative bond, as reported by Gillingham's group.^36^ This strategy can be further used for rapid oxime formation, which is 6 orders of magnitude quicker than without the boronic acid moiety.^37^ In contrast, hydrazines and semicarbazides reveal remarkable stability through a diazaborine product (*vide infra*).

**Table 1:** Summary of reversible chemistry on parenting iminoboronate and their applications

Electrophile	Nucleophile	p*K*a	Disso. const. (*K*_d_)	Applications	Ref.
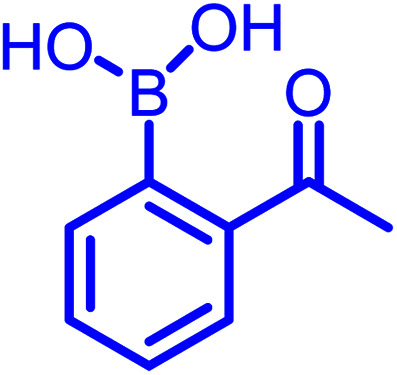	Primary amine	9–11	∼10–17 mM	Labelling of bacterial membrane; covalent drug design	19–22
	Hydrazide	5–6	∼0.6 mM	Not seen yet	35
	Oxyamine	4–5	∼0.14 × 10^−1^ mM	Not seen yet	35
	Phenylhydrazine	5.2	∼0.7 × 10^−4^ mM	Protein labelling	35
	Cysteine	8.3	∼0.5 mM	Not seen yet	45
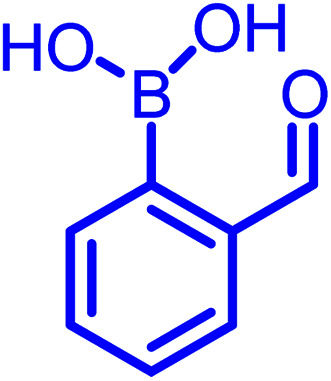	Primary amine	9–11	∼10 mM	Self-healable hydrogels	30,33,34
	Hydrazide	5–6	Not determined	Semicarbazide detection	41,57
	Oxyamine	4–5	∼0.38 × 10^−5^ mM	Not seen yet	36
	Cysteine	8.3	∼0.5 × 10^−2^ mM	Protein labelling, Cys scavenging	43,44,46
	l-Dap	6.7	∼0.1 mM	Change in Cys in blood serum	48
	Tris	8.1	0.1 mM	Peptide cyclization	42

In the past decade, many groups^35,37^ investigated the kinetics of α-nucleophiles reacting with 2-FPBA/2-APBA at neutral pH, which is exceptionally rapid (>10^3^ M^−1^ s^−1^) because of the self-catalysis performed by the *ortho*-boronic acid in the rate-determining step (Fig. 2a). We have collectively inspected through the literature and found an inverse relationship between the p*K*_a_ of nucleophiles and the thermodynamic stability of the respective iminoboronate conjugates. The higher p*K*_a_ of a nucleophile correlates with a lower stability in a spontaneous dissociation. On the other hand, 2-FPBA conjugates enjoy better thermodynamic stability due to the weaker strength of the Lewis conjugate (N → B dative bond, or solvent insertion) in comparison to 2-APBA conjugates.

### 2.2 Kinetically labile

**Fig. 6:**
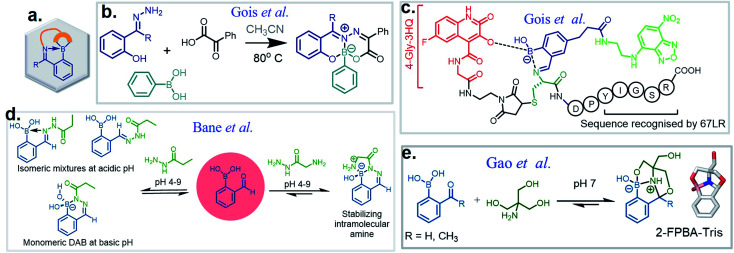
Modules of kinetically stable iminoboronate: (a) proposed cartoon for the strategy, (b) three-component assembly to stabilize iminoboronates, (c) incorporation and application of BHS, (d) conjugation reaction between 2-FPBA and hydrazides. Conjugate with the amine derivative of hydrazide realize highly stable conjugate in the application of protein labelling, (e) tris base conjugation with 2-FPBA.

In this section, we discuss the thermodynamically stable but kinetically labile iminoboronate products, in which a synergistic influence of structural effects cooperate to stabilize iminoboronate products (Fig. 6a) for applications in bioconjugations and medicinal chemistry. For example, a novel advance by the Gois group involved a high yielding and diastereoselective one-pot assembly reaction of three components to identify enzyme inhibitors.^38^ They extended this work to the synthesis of boronic acid salicylidenehydrazone (BASHY) dyes,^39^ which were reported to be stable, non-cytotoxic, and highly fluorescent. The conjugation strategy was subsequently applied to develop live-cell imaging methodologies. The components involved were hydrazones of various phenyl glyoxylic acids, *N*,*N*-diethyl salicylaldehyde and phenylboronic acid (PBA) derivatives (Fig. 6b). A three-component assembly yielded dyes that exhibited polarity-sensitive green-to-yellow emission with quantum yields up to 0.6 in non-polar environments. Further, the dyes entrapped in poly(lactide-*co*-glycolide) (PLGA) nanoparticles (NPs) were readily internalized by dendritic cells and were able to selectively stain lipid droplets in HeLa cells; an effect which was comparable to the archetypal Nile red dye.

Very recent work by this group was aimed at directing iminoboronate formation with specific amino acid side chains by installing a ‘boron hot spot’ (BHS) at desired sites on a peptide.^40^ 4-Carboxamido 3-hydroxyquinolin-2(1*H*)-one with a glycyl linker at the amide NH_2_ (4-Gly-3HQ), having a hydroxyl group that can coordinate to boron atom, was chosen as the BHS with an affinity of *K*_a_ = 699 ± 2 M^−1^ for PBA (Fig. 6c). The BHS, having a maleimide group, was installed on a peptide containing an N-terminal Cys residue, a step that also allowed its reversibility in the presence of glutathione. They reported the reaction of BHS–Cys with 2-FPBA (20 mM, pH 7) to proceed with a *K*_d_ of 1.7 × 10^−2^ mM at 37 °C. An important aspect of this work is that iminoboronate formation only occurs with the N-terminal amine even in the presence of other residues. *C*-Ovalbumin-BHS containing N-terminal amine and ε-amine of Lys was subjected to 1000 eq. of 2-FPBA overnight and the resulting double iminoboronate upon dilution only yielded the N-terminal iminoboronate, thus proving the favourability of the reaction. Utilizing this chemistry, they developed a cleavable fluorescent probe conjugated to a laminin fragment. Laminin is recognized by 67LR, a receptor that is over-expressed in cancer cells. They demonstrated that the probe, forming an iminoboronate with 2-FPBA, was readily internalized into HT29 cancer cells, probably through a BA-assisted permeation, thus demonstrating the utility of 67LR as a promising target for delivery of cargo.

Over the past few years, several groups have worked to discover highly stable iminoboronate conjugates by exploiting boronic acid accelerated kinetics to reveal opportunities for bioconjugation. Some strategies do not retain the iminoboronate skeleton as a final product. For example, Bane and co-workers investigated the reaction with hydrazides and α-amino hydrazides in 2017 (ref. 41) following their work with hydrazines. They concluded that the reaction of 2-FPBA with propanoic acid hydrazide in aqueous solutions (2 mM final concentrations) yielded a mixture of *cis* and *trans* hydrazone products at pH 4, however, a diazaborine (DAB) product at pH 9. In the quest to obtain monomeric DAB products, they used α-amino hydrazides as the nucleophile partner, anticipating the neighbouring intramolecular amine group to provide a coordinating partner to the boron atom (Fig. 6d). The product obtained was indeed stable over pH 4–9, as confirmed by ^1^H NMR spectroscopy. They demonstrated the utility of this reaction by fluorescently labelling BSA incorporated with hydrazide and α-amino hydrazides, followed by rapid gel filtration and SDS-PAGE analysis, where only the BSA modified with α-amino hydrazides withstood SDS-PAGE conditions.

Tris base, widely used as a component of buffer solutions, was found to form oxazolidinoboronate (OzB) complexes (Fig. 6e) with 2-FPBA/APBA. These adducts possess fast kinetics of formation and higher chemical stability as compared to the conjugates of cysteine and 1,2-diaminoproprionic acid (discussed in the next section).^42^ The stability of the products was revealed by their crystal structures. The favourable binding of 2-FPBA to the hindered amines was also reflected with a *K*_d_ of 10^−4^ M as compared to 2-APBA (10^−3^ M). Although reversibility with Cys occurs, nevertheless FPBA–Tris conjugation (0.5 mM each) in *E. coli* proceeded to 70% completion, thus showing its excellent biocompatibility. They further applied this strategy to cyclize peptides and demonstrated the stability of the complex in 0.5 mM H_2_O_2,_ thus validating its use in peptide therapeutics.

### 2.3 Repurposed

**Fig. 7:**
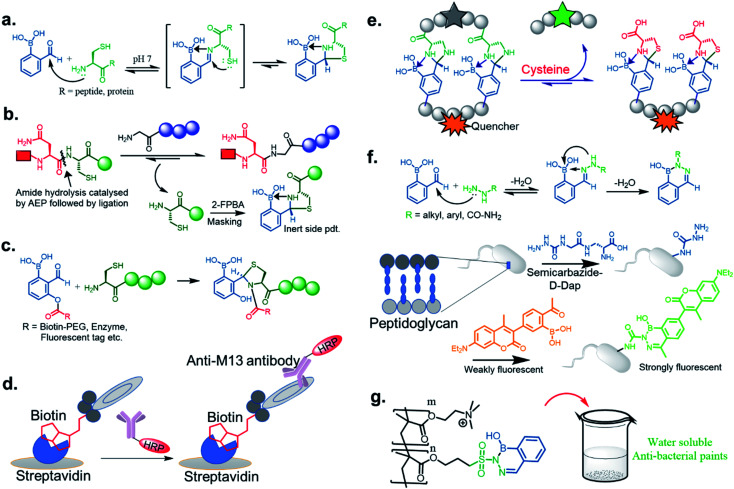
Modules of repurposed conjugates *via* iminoboronate chemistry: (a) mechanism of thiazolidino boronate formation, (b) scavenging Cys in AEP labelling, (c) acyl transfer technology to kinetically inert thiazolidine formation *via* thiazolidinoboronate, (d) modification of M13 phage library by acyl transfer technology to stable Thz formation, (e) schematic presentation of imidazolidino boronate exchange to thermodynamically more stable conjugate thiazolidino boronate by Cysteine. (f) Stable diazaborine formation applied to bacterial peptidoglycan modification for selective labelling. (g) Utilization of diazaborine derivative as an active component in water-soluble anti-bacterial paint.

We now examine how investigators have repurposed the iminoboronate formation by trapping the dynamic imines through intramolecular nucleophilic addition. In other words, the final product does not possess the iminoboronate structure. This approach generates kinetically inert, thermodynamically stable heterocycles under a pH range of 6–10. Initially, the Gao^43^ group, and at the same time the Gois^44^ group, independently reported such chemistry through thiazolidinoboronate (Thz) formation (Fig. 7a) where an N-terminal cysteine of peptides and proteins readily conjugated to 2-FPBA to form a stable tricyclic core. The proximity driven attack by the thiol group from the cysteine iminoboronate leads to the formation of a stable complex with a *K*_d_ ∼ 5 μM with 2-FPBA.^45^ The reaction was shown to be rapid (*k*_2_ ∼ 10^3^ M^−1^ s^−1^), efficient, diastereoselective, and competed well against the other free thiol groups (*e.g.*, GSH) present in the reaction media. The 2-FPBA–Cys conjugate did show interference from endogenous free cysteine molecules, but reaches equilibrium with cysteine in an hour time scale. Overall, the complex shows dissociation and attains a quick equilibrium upon acidification below pH 5.5. Only about 50% product dissociation occurs at pH ∼3, which indicates incomplete dissociation of this conjugate at mildly acidic pHs. Such a feature is promising for recombinant protein drug conjugation and delivery in a mildly acidic pH milieu. Recently Tang *et al.*^46^ exploited this reaction for scavenging cysteine by-products in the labelling of proteins and peptides by asparaginyl endopeptidases (AEPs). Asn–Cys–Leu was used as the recognition sequence for AEP, and the Cys–Leu generated by-product was scavenged by 2-FPBA leading to excellent yields of the otherwise reversible AEP labeling (Fig. 7b). This modification highlights the use of such a chemo-enzymatic labelling protocol. In contrast, the 2-APBA conjugate with Cys is more dynamic in nature, with a *K*_d_ ∼ 0.5 mM, likely due to steric interactions. Thus, the keto derivative conjugates may be more suitable for DCL purposes. A very recent paper by Li *et al.* established a kinetically inert N-terminus cysteine conjugate *via* acyl transfer to the Thz product (Fig. 7c). The kinetics for biomolecule conjugation they explored with this chemistry (*k*_2_ ∼ 5000 M^−1^ s^−1^) is a few orders of magnitude faster than the existing N-terminus cysteine conjugations.^47^ This robust conjugation method allowed them to demonstrate stable enzyme labelling, and to access a wide chemical space in a phage display library (Fig. 7d).

Although serine does not yield such a kind of conjugate, the rapid and reversible formation of imidazolidino boronate between 2-FPBA and 1,2-diaminoproprionic acid (Dap) has been noticed in Gao Lab.^48^ Imidazolidino boronate, as derived from Dap, was found to be 20 times less stable (*K*_d_ ∼ 0.1 mM) than Thz, which therefore can exchange in the presence of free Cys. This discovery was applied to the monitoring of changes in Cys concentrations in blood serum in real-time (Fig. 7e). Overall, the observations reveal that the side chain p*K*_a_'s of amino acids (Ser, Cys, and Dap) control the thermodynamic stability of conjugates.

The iminoboronate products of hydrazine and semicarbazide are kinetically trapped, but now by the formation of a stable, aromatic diazaborine. The aromatic ring is formed by the NH group adjacent to the imine nitrogen forming a bond with the boron atom in a second dehydration step (Fig. 7f). The facile propensity for heterocycle formation with hydrazines is presumably explained by the electron density of the α-N, while hydrazides have lower density due to resonance with a carbonyl.^35,41^ Diazaborines are therapeutically very important scaffolds, having antibacterial activity. They were reported as early as 1964.^49^ Högenauer and Woisetschläger^50^ have revealed their mode of action by inhibiting lipopolysaccharide biosynthesis on Gram-negative bacteria. This rapid diazaborine formation under physiological conditions has been used to develop protein conjugation and new biorthogonal reactions by the Bane^51^ and Gao groups.^45^ Remarkably, 2-APBA and semicarbazide were demonstrated to be a “sweet partner” in developing ideal biorthogonal reactions formation as reactants and product do not exhibit toxicity, and the reaction can be used for selective bacterial detection (Fig. 7f) *via* peptidoglycan modification of fluorogenic probe^52^ or fluorophore-labeled conjugates.^45^

Recently, Kocak *et al.*^53^ reported the synthesis of 3-((1-hydroxybenzo[*d*][1,2,3]diazaborinin-2(1*H*)-yl)sulfonyl)propyl methacrylate (DAZBMA), as well as a polymer thereof with 2-dimethylaminoethyl methacrylate. The polymer was used as an antibacterial and anti-quorum-sensing material when combined with 2-FPBA (Fig. 7g). Interestingly, this copolymer could be used in water-soluble paints to reduce pathogenicity, and thus can be used in hospitals. In addition, Scott *et al.*^54^ synthesized boron-containing diazaborines and thiosemicarbazones from 4-ethyl-3-thiosemicarbazide and 2-FPBA derivatives to have a better understanding on their antimicrobial activity. Three compounds showed promising antifungal activity against *S. cerevisiae*, which was comparable to amphotericin B, while antibacterial activity against *B. cereus* was only observed for one compound (Table 2). In recent years, this diazaborine scaffold has also found use to generate fluorescent molecules for sensing anions^55^ and serine protease inhibitors.^56^ Semicarbazide is a toxic food contaminant and is widely found in foodstuffs. Kong and co-workers^57^ developed a simple method to determine trace amounts of semicarbazide. They labelled the contaminant with 2-FPBA for 7 min at room temperature and confirmed it by HPLC with fluorescence detection. By applying mass spectrometry, they achieved a detection limit of 0.36 μg L^−1^ and quantitation limits of 1.17 μg L^−1^ with high accuracy.

**Table 2:** Antimicrobial activities of novel boron-containing diazaborines and thiosemicarbazones reported by Scott *et al*

Compounds	*Saccharomyces cerevisiae* (fungi)				*Bacillus cereus* (Gram-positive bacteria)		
	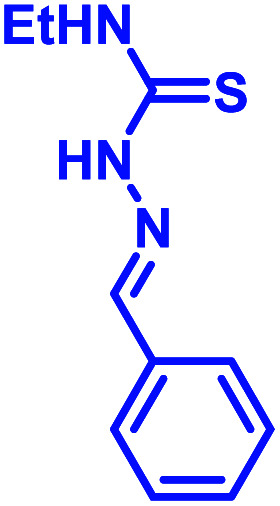	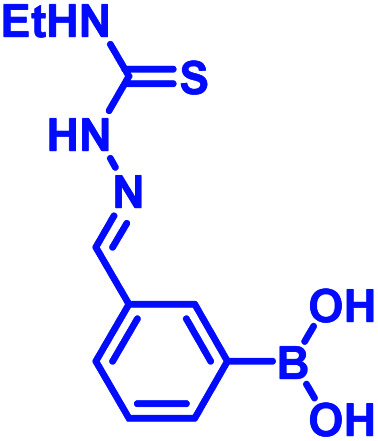	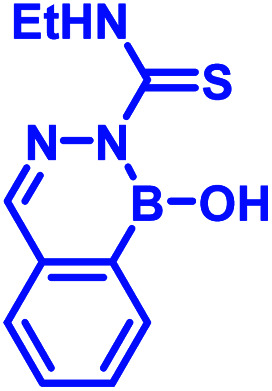	Amphotericin B (control)	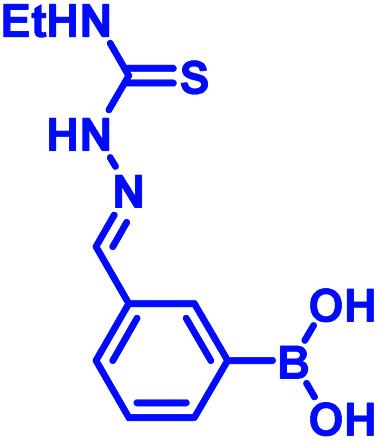	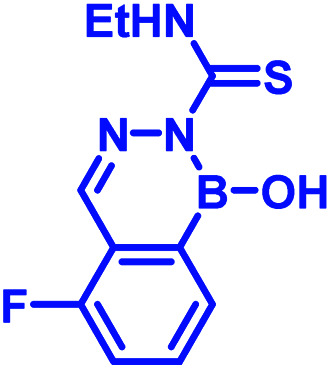	Erythromycin (control)
Dose (μg per disk)	100	100	100	100	100	100	15
Clear zone (mm ± SD)	3.9 ± 0.6	4.0 ± 0.8	4.3 ± 0.8	4.1 ± 0.2	3.5 ± 0.5	3.4 ± 0.2	13.5 ± 1.0

Shimo *et al.*^58^ utilised the DAB moiety to develop a probe for the determination of the absolute configuration of mono-alcohols. The probe, containing a tri-coordinated boron species (^11^B NMR: *δ* = 27 ppm), was obtained by mixing 2-FPBA and acridine hydrazine. It was allowed to react with a chiral alcohol to obtain a tetra-coordinated borate species (^11^B NMR: *δ* = 5.7 ppm) with imine nitrogen of the acridine moiety. From the crystal structures, it was evident that higher *dr* value was obtained from the interaction of the structurally different groups of chiral secondary alcohols with the large π-face of the acridine moiety. Interestingly, selective recognition with alcohols was achieved with no interference from amine or thiol groups.

**Fig. 8:**
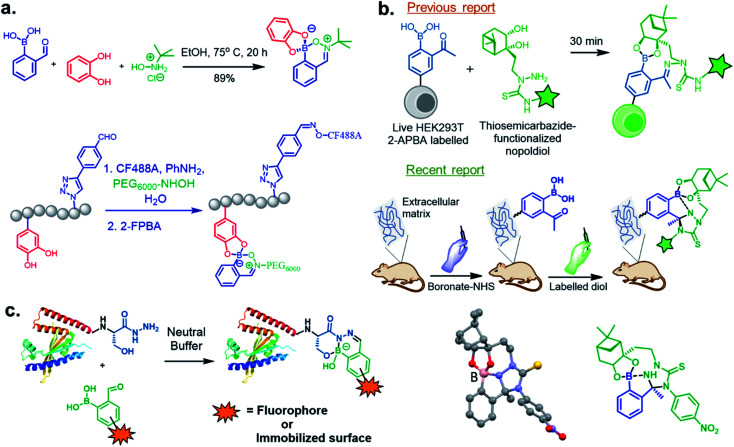
(a) Three component assembly applied to dual-labelling of a peptide, (b) live cell imaging by Thz-modified nopoldiol, and recently reported tetracyclic adduct with *in vivo* applications to mice. (c) β-Hydroxy stabilized B–N heterocycles for efficient C-terminal protein modification.

In 2017, Meadows *et al.* also disclosed the formation of an irreversible, three-component assembly with 2-FPBA, catechol, and *N*-hydroxylamine in aqueous media.^59^ The complex once formed, was stable in neutral aqueous conditions for over 72 h and only the catechol group was replaced with hydroxyl groups at pH 13 while the hydroxylamine addition was stable to acid, base, and heat for 24 h. This click reaction was used in the dual-labelling of a peptide (Fig. 8a). Working on similar lines, Hall and co-workers designed a synergic system having 2-APBA and thiosemicarbazide-functionalized nopoldiol (Fig. 8b).^60^ The click reaction proceeded with a rate constant of 9 M^−1^ s^−1^ in aqueous solution while also remained unaffected by biological diols and aldehyde electrophiles. They demonstrated live-cell imaging of HEK293T cells using a SNAP-tag approach. Recently they communicated an *in vivo* proof-of-concept study using the same biorthogonal reaction. Through crystallography, they established that the conjugation proceeds *via* double condensation of arylboronate and nopoldiol leading to a tetracyclic adduct. Interestingly, the reaction was shown to work in a live animal setting.^61^

Following their previous report on the stable conjugates between α-amino hydrazides and 2-FPBA/2-APBA, the Bane group reported the formation of highly stable products from the reaction of 2-FPBA with β-hydroxy hydrazides.^62^ An apparent second-order rate constant for the hydrazone formation step was calculated to be ∼955 M^−1^ s^−1^, and the combined ring closure event was ∼0.014 s^−1^. They demonstrated this strategy should generally be applicable for rapid, efficient site-specific protein labelling, protein immobilization, and preparation of highly pure functionalized proteins (Fig. 8c).

## 3. Salicylhydroxamic–boronate (SHAB) conjugate

**Fig. 9:**
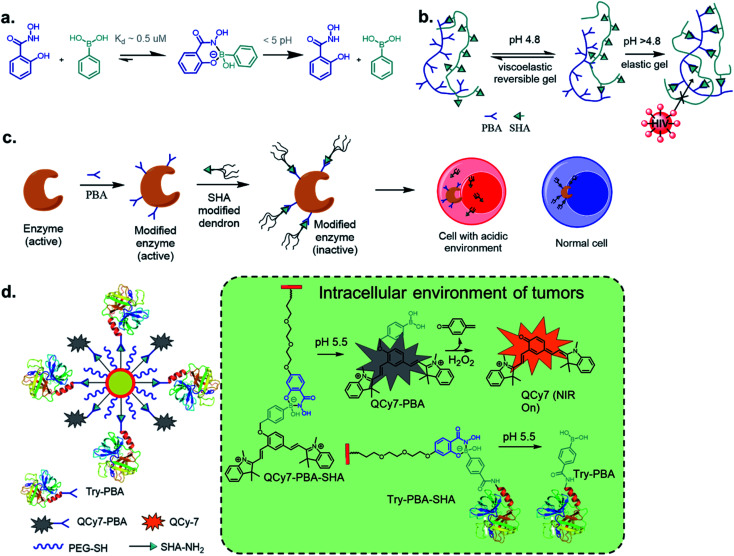
(a) Dynamic click reaction between SHA and PBA and stimuli-triggered dissociation of the SHAB conjugate in biological environments enable (b) HIV-barrier gel, (c) precision biotherapeutics by controlling enzyme activity, (d) protein delivery as well as turn-on NIRF reporter.

Perhaps, the reaction of BA with salicylhydroxamic acids (SHA) has garnered the most attention besides that of boronate ester formation with diols. Discovered by Stolowitz and co-workers^63^ in 2001, it has been applied extensively, and a detailed thermodynamic study was reported by Martínez-Aguirre *et al.*^64^ in 2018. With an association constant of 10^4^ M^−1^ at pH 7.4 for the complex of phenylboronic acid (PBA) and SHA, the complex is stable at physiological pH, undergoing possible hydrolysis at pH < 5 (Fig. 9a). The kinetics of this conjugation has yet to be determined.

Stolowitz and co-workers have applied this conjugation method for protein (modified with PBA) purification by functionalizing SHA to Sepharose beads.^63^ The conjugate showed strong and selective formation, and was resistant to successive washing by basic and neutral eluents. The conjugate, however, can be disrupted at pHs < 3, as revealed by the successful elution of the protein. Further, the thermodynamic stability of the conjugate could be increased by including two BA groups on the phenyl ring.^65^ Jaffrey and co-workers demonstrated the biorthogonality of this click reaction *via* assembling multiple small molecules and peptide dimers in a cellular environment as a means to create agonists for thrombopoietin receptor c-Mpl.^66^ Thus, early in the current decade, this stimulus-responsive click reaction proved to be efficient for several bioengineering purposes.

Analogously, Cristiano and co-workers demonstrated tumour-targeted gene therapies for use in the clinical treatment of cancer using the SHAB click reaction.^67^ They employed a targeted delivery of a DNA cationic-polyplex using a non-viral approach with the CNGRC peptide. This peptide is selective to the CD13 receptor, which is over-expressed in tumour cells. It was linked to a PEI–DNA polyplex *via* a phenyl diboronic acid–SHA complex, while also maintaining the overall integrity of the vector. The SHAB conjugate has also been used in the construction of smart hydrogels by Weil's group, encapsulating cytochrome *c*, a proapoptotic enzyme that induces apoptosis in the acidic environment of tumour cells. The hydrogels, employing PBA–SHA linkages, show interesting rheological and self-healing properties.^68^

One novel application of SHAB click chemistry is in the developing of HIV-barrier gels.^69–71^ The diffusion of HIV virions from seminal fluid to vaginal mucosa occurs during insemination when the pH of the vaginal fluid increases. To exploit this as a means to slow HIV infections, the Kaiser group envisioned employing loose gels that become increasingly viscous during insemination because this would decrease the virion diffusion. An increase in the affinity of PBA–SHA binding with increasing pH was utilized. The synthesized co-polymers underwent high sol–gel transitions at low pH while limiting virion diffusion at pHs > 4.8 at a rate sufficient for natural vaginal mechanisms to deactivate infectious virions (Fig. 9b).

The Weil's group has explored precision biotherapeutics utilizing SHAB conjugates. They incorporated dendrimers onto active enzymes such as trypsin, papain, and DNase I, which rendered these biomolecules inactive. Upon exposure to acidic conditions in the desired cellular environment, the biomolecules regained their catalytic activity due to the rupture of the dendrimeric shell (Fig. 9c).^72^ The same group reported ‘tag and modify’ protein conjugation, in which they modified a desired protein by installing PBA, followed by purification using carbohydrate-based column chromatography. The protein was further fluorescently tagged by SHA–BODIPY, all while retaining its enzymatic activity.^73^ Using similar chemistry, Pei *et al.* constructed a near-infrared fluorescence (NIRF) probe and a protein bound to a single nanoparticle (NP). The probe was attached by a benzyl ether linkage to PBA and coordinated to SHA–PEG, while the protein modified with PBA was also coordinated to SHA–PEG.^74^ The NPs were efficiently taken up by A549 cells, and the acidic environment disrupted the PBA–SHA conjugate leading to the release of both protein and probe, whose turn-on NIRF was observed upon oxidation by H_2_O_2_. Analogous work was recently reported by Liu *et al.* where polymer–protein nano-assemblies was prepared through SHA–PBA chemistry and delivered to tumour environments by biologically triggered oxidation by H_2_O_2_.^75^

**Fig. 10:**
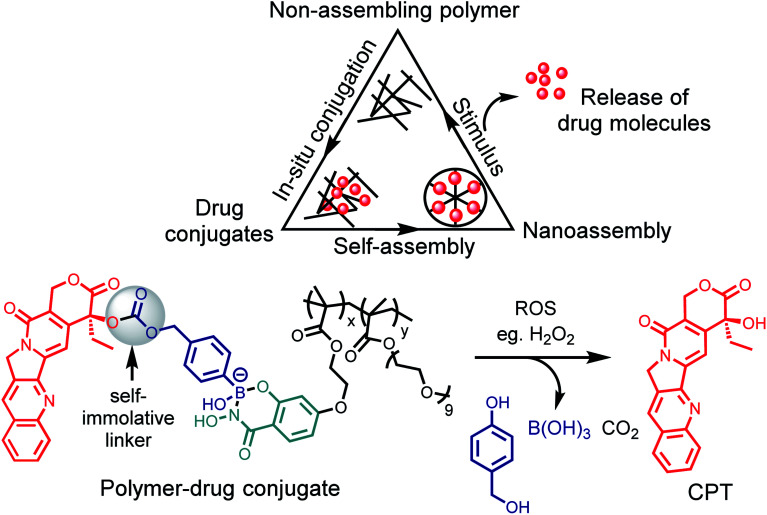
Post-polymerization functionalization strategy and a scheme for ROS triggered oxidation to drug release.

The recent work of Thayumanavan *et al.* have demonstrated the utility of this reaction in a post-polymerization functionalization strategy.^76^ The synthesized hydrophilic polymer bearing SHA functionality did not show any self-assembly or formed ill-defined polymer aggregates (Fig. 10). Upon addition of the hydrophobic drug bortezomib, which contains a BA functionality, *in situ* formation of nanoassemblies with a size ∼ 50 nm was observed. The polymer–drug conjugate nanoassemblies showed stable release kinetics at pH 5, as evaluated with three different cancer cell lines HeLa, MDA-MB-231, and MCF-7. They also demonstrated the use of a self-immolative linker for administering drugs in their original form. The anti-cancer drug camptothecin was modified with a BA moiety that self immolates only under oxidizing conditions to release the original drug from the polymer. Pieszka *et al.* demonstrated the formation of controlled supramolecular assembly inside living cells using bimodular peptide sequences.^77^ They utilized SHAB chemistry to link the bimodular peptide with cell-penetrating TAT peptide, which upon entering A549 cells dissociated under the acidic environment. The complexity of peptide assembly was dictated by the microenvironment of the cell. This assembly was shown to induce apoptosis, thus broadening the concept of influencing cellular functions by structure formation.

## 4. Summary and future outlook

The aforementioned examples summarize the last five years of significant advancements in the use of iminoboronate chemistry, followed by SHAB chemistry. We described that the fast reaction kinetics of exchange could in some cases be beneficial, while in other cases, the reversibility of iminoboronate due to the Lewis acidic nature of BA under physiological conditions is detrimental. While the hydrolysis may be useful for traceless applications, it can also be beneficial to construct multicomponent-assembled iminoboronate drug conjugate. The spontaneous reversibility is advantageous in designing covalent drugs, targeted probes for biomolecules, self-healable biopolymers, and DCLs. Alternatively, the rapid kinetics of iminoboronate formation that can be kinetically trapped at physiological pH, suggests applications suited for bioconjugation, labelling, and biorthogonal reporters. Further, stimuli-responsive iminoboronate technologies are of foremost interest for developing biomaterials, biopolymers, and drug delivery vehicles. While SHAB chemistry formation rates are lower in comparison, several designs can lead to research breakthroughs for designing sensors, biorthogonal reporters, and antibody–drug conjugates. The basic principles of SHAB chemistry allow for biomaterials encapsulated drug delivery in a lower pH biological milieu.

Clearly, iminoboronate- and SHAB-mediated chemistries are still under rapid development for *in vivo* applications, and we anticipate several future directions for these fields. Using only BA probes, achieving target selectivity and minimizing related toxicity are the significant challenges, because of BAs are known to form ester conjugates with endogenous carbohydrate diols. Similarly, 2-APBA/2-FPBA probes form iminoboronate complexes in an uncontrolled manner to endogenous amines, consequently, risking off-target modifications. Favorably, as the conjugations are reversible, they should be readily used as covalent probes by attaching with ligands^3^ that are specific to a target; such as how the covalent drugs Bortezomib and Ixazomib act. Further, multicomponent reversible conjugates of BAs may be anticipated as prodrug formulations. Likewise, diazaborine derivatives, inert to endogenous nucleophiles, are a potential scaffold for small molecule inhibitor discovery. Diazaborines need to be further explored and examined for *in vivo* studies. We also speculate that SHAB chemistry can further be modified to form a kinetically stable conjugate for the development of BA-mediated biorthogonal chemistries for diagnostics development. Importantly, these two chemistries have found wide multidisciplinary science applications, and the understanding of their basic principles documented here is anticipated to fuel future applications.

## Conflicts of interest

The authors declare no conflict of interest.

## Supplementary Material
